# Clinical Characteristics of Patients with Intra-Abdominal Infection Caused by *Stenotrophomonas maltophilia*

**DOI:** 10.3390/jcm14113974

**Published:** 2025-06-04

**Authors:** Chien-Liang Chen, Chun-Chou Tsai, Wei-Ping Chen, Feng-Yee Chang, Ching-Mei Yu, Hung-Sheng Shang, Leung-Kei Siu, Ya-Sung Yang, Jung-Chung Lin, Ching-Hsun Wang

**Affiliations:** 1 Division of Infectious Diseases, Department of Internal Medicine, Zuoying Armed Forces General Hospital, Kaohsiung 81300, Taiwan; vecear@gmail.com; 2 Division of Infectious Diseases and Tropical Medicine, Department of Internal Medicine, Tri-Service General Hospital, National Defense Medical Center, Taipei 11490, Taiwan; seabrotherba@yahoo.com.tw (C.-C.T.); tkjh10026@hotmail.com (W.-P.C.); fychang@mail.ndmctsgh.edu.tw (F.-Y.C.); ysyoung4097@gmail.com (Y.-S.Y.); linjungchung1@yahoo.com.tw (J.-C.L.); 3 Division of Infectious Diseases, Department of Internal Medicine, Taoyuan Armed Forces General Hospital, Taoyuan 32551, Taiwan; 4 Department of Pathology, Tri-Service General Hospital, National Defense Medical Center, Taipei 11490, Taiwan; sheep565656@gmail.com (C.-M.Y.); iamkeith@tsghms.ndmctsgh.edu.tw (H.-S.S.); 5 Institute of Infectious Diseases and Vaccinology, National Health Research Institutes, Miaoli 35053, Taiwan; klksiu@gmail.com

**Keywords:** intra-abdominal infection, *Stenotrophomonas maltophilia*, mortality, risk

## Abstract

**Background:** Intra-abdominal infections (IAIs) caused by *Stenotrophomonas maltophilia* have rarely been reported. This study aimed to describe the clinical characteristics and risk factors for mortality among patients with *S. maltophilia* IAIs. **Methods:** A retrospective study was conducted on inpatients with IAIs caused by *S. maltophilia* at Tri Service General Hospital from 2004 to 2017. Clinical and microbiologic data of the included cases were reviewed via medical charts and microbiology databases. Multivariable logistic regression analyses were performed to identify risk factors for in-hospital death. **Results:** In total, 110 patients were diagnosed with *S. maltophilia* IAIs. Malignancy (56.3%) and liver cirrhosis (35.3%) were the most commonly identified underlying diseases. The major causes of *S. maltophilia* IAIs were biliary tract infection (42.7%), recent abdominal surgery (35.4%), and spontaneous bacterial peritonitis (20.0%). Polymicrobial infections were observed in 84 (76.4%) patients. In addition to *S. maltophilia*, co-cultured bacteria (*n* = 140) included *Enterobacterales*, representing 19.3% (27/140) of the total isolates, and non-fermentative aerobes, comprising 29.3% (41/140). In addition, anaerobic bacteria and fungi accounted for 9.2% (13/140) and 10% (14/140), respectively. The overall mortality rate was 40.9%. Multivariable logistic regression analysis revealed that high Sequential Organ Failure Assessment scores and malignancies were independent risk factors for mortality, while the immediate administration of appropriate antibiotics targeting *S. maltophilia* was a protective factor (*p* < 0.05). **Conclusions:** Patients with an underlying malignancy or liver cirrhosis were at risk for IAIs caused by *S. maltophilia*. The prompt initiation of effective antibiotics against *S. maltophilia* is critical for achieving favorable outcomes.

## 1. Introduction

*Stenotrophomonas maltophilia*—formerly known as *Pseudomonas* or *Xanthomonas maltophilia*—is an aerobic Gram-negative bacillus that can survive and be isolated from a wide variety of aqueous environments [1,2]. In recent years, *S. maltophilia* has emerged as a significant nosocomial pathogen due to its ability to cause various infections in immunocompromised inpatients [3,4]. The World Health Organization lists *S. maltophilia* as an important Gram-negative multidrug-resistant bacterial pathogen in hospitals [5]. Various types of infections caused by *S. maltophilia* have been reported, with pneumonia being the most common, followed by bloodstream infections, while other infections are less frequent [1]. Previously reported risk factors for acquiring *S. maltophilia* infection include prolonged hospitalization, the retention of intravascular catheters, the use of broad-spectrum antibiotics, extended mechanical ventilation, and underlying conditions such as neutropenia caused by malignancy [1,5]. Treating *S. maltophilia* infections is challenging due to this bacterium’s intrinsic and acquired resistance to a wide range of antibiotics. Several molecular antibiotic resistance mechanisms have been reported in *S. maltophilia*, including β-lactamase production and the presence of efflux pumps, as well as integrins carrying a variety of gene cassettes encoding antibiotic resistance [6,7,8]. Despite low virulence, a considerable attributable mortality rate (up to 37.5%) and overall mortality (69%) due to *S. maltophilia* infections have been reported [9,10].

Intra-abdominal infections (IAIs) represent a broad spectrum of infectious processes, including peritonitis, intra-abdominal abscesses, appendicitis, and hepatobiliary infections. These infections are often polymicrobial in nature, with pathogens originating primarily from the gastrointestinal flora. Among the various types of infection caused by *S. maltophilia*, intra-abdominal infections (IAIs) have been rarely reported due to their lower prevalence. The Study for Monitoring Antimicrobial Resistance Trends (SMART) surveillance program, conducted in China from 2002 to 2009, investigated the causative pathogens of IAIs [11]. The most frequently isolated organisms were members of the *Enterobacteriaceae* family, particularly *Escherichia coli* and *Klebsiella pneumoniae*. *S. maltophilia* accounted for only 1.5% of the aerobic and facultative Gram-negative bacilli recovered from IAI specimens in the SMART study. Due to the low prevalence of intra-abdominal infections (IAIs) caused by *S. maltophilia*, the available literature is limited to case reports or small case series. These reports suggest that *S. maltophilia* IAIs typically occur in immunocompromised individuals, such as those with malignancies, organ dysfunction, or receiving immunosuppressive therapy [12,13,14]. Some cases have also occurred after abdominal surgery and/or are associated with prior antibiotic exposure [12,13,14]. Gastrointestinal colonization or mucosal disruption has also been implicated as a potential endogenous source [12]. However, due to the limited number of cases, it remains difficult to draw comprehensive conclusions about the role of *S. maltophilia* in patients with IAIs.

The purpose of this study was to analyze the clinical characteristics and risk factors for mortality in patients with IAIs caused by *S. maltophilia* from a single center between 2004 and 2017, with the goal of providing a clearer understanding of this rare clinical entity.

## 2. Materials and Methods

### 2.1. Study Design and Data Collection

This longitudinal observational study was conducted from 2004 to 2017 at the Tri-Service General Hospital, a tertiary referral center in northern Taiwan. The research ethics committee of Tri-Service General Hospital reviewed and approved the study (TSGHIRB approval number: 2-101-05-074, 9 February 2018). Hospitalized adult patients with their first episode of *S. maltophilia* isolated from various clinical specimens between January 2004 and December 2017 were initially identified using a computerized microbiologic database. Cases with *S. maltophilia* isolated from abdominal specimens were further reviewed through the hospital’s electronic medical record (EMR) system to identify intra-abdominal infections. Patients were defined as having *S. maltophilia* IAIs if they had a positive *S. maltophilia* culture from an abdominal specimen and clinical symptoms consistent with sepsis [15], or if evidence of an intra-abdominal infection was observed during an operative procedure or imaging studies (such as CT or ultrasound) based upon the medical records. Patients younger than 18 years, those not admitted to the hospital, and those without clinical signs consistent with sepsis were excluded. Specimens of abdominal origin were collected using aseptic procedures from ascitic fluid via paracentesis, aspirates from the drainage tube of the intra-abdominal cavity, or samples from the abdominal viscera (liver, gallbladder, spleen, and pancreas). Relevant clinical data on the identified patients, including age, gender, underlying diseases, disease severity, antibiotic treatment, and outcomes were also recorded via the EMR system. The different occurrence locations of IAIs caused by *S. maltophilia* were categorized as community-acquired, hospital-acquired, and healthcare-associated, based on previously reported criteria [16]. **Hospital-acquired infection** was defined as an infection diagnosed ≥48 h after hospital admission, with the time counted from the first admission date of the transferred patients. **Healthcare-associated infection** was defined as an infection present at the time of admission or within 48 h of admission in patients who met at least one of the following criteria: (1) received intravenous therapy, wound care, or specialized nursing care at home within the previous 30 days; (2) attended a hospital or hemodialysis clinic or received intravenous chemotherapy in the prior 30 days; (3) had been hospitalized in an acute care facility for ≥2 days within the past 90 days; or (4) resided in a nursing home or long-term care facility. **Community-acquired infection** was used to define infections that did not meet any of the above healthcare-associated criteria. The Charlson comorbidity index was calculated based on the collected comorbidities [17]. The severity of patients was assessed using the Sequential Organ Failure Assessment (SOFA) score [18]. Neutropenia was defined as an absolute neutrophil count (ANC) of less than 1500 cells/μL at the time of sepsis onset. Septic shock was defined by the presence of systolic blood pressure lower than 90 mmHg or when inotropic agents were required to maintain blood pressure with evidence of organ hypoperfusion [15]. In-hospital mortality was defined as death for any cause after IAI onset at any time within current admission, and 30-day mortality was defined as death within 30 days of the index culture. Previous antibiotic exposure was defined as the administration of any systemic antibiotic within 14 days prior to the onset of *S. maltophilia*-related IAIs. Hospital stay before isolation was defined as the interval between the date of hospital admission and the date of *S. maltophilia* isolation from abdominal specimens associated with sepsis onset. Other concomitant pathogens isolated from the same specimens were also documented. Appropriate antibiotic therapy was defined as the administration of at least one antimicrobial agent to which the *S. maltophilia* isolate was susceptible, given within 48 h of sepsis onset. The infection control measure was defined as the performance of either percutaneous drainage or surgical debridement to manage *S. maltophilia*–related IAIs.

### 2.2. Microbiology Examination

Clinical specimens of abdominal origin were submitted to the microbiology laboratory for aerobic, anaerobic, and fungal cultures. For aerobic cultures, colonies were grown on Trypticase Soy Agar with 5% sheep blood (Nippon Becton Dickinson, Tokyo, Japan), MacConkey Agar, and Columbia CNA Agar (Creative Microtech Corp., New Taipei City, Taiwan) under standard incubation conditions. For anaerobic cultures, specimens were plated onto CDC Anaerobe Blood Agar (Nippon Becton Dickinson, Tokyo, Japan), Anaerobic Base Plate, and KVLB Agar (both from Creative Microtech Corp., Taiwan). Cultures were incubated in an anaerobic workstation (Concept Plus, BAKER RUSKINN, Bridgend, UK) under a gas mixture composed of 10% CO_2_, 10% H_2_, and 80% N_2_. For fungal cultures, specimens were inoculated onto ICG Agar, Chromogenic Candida Agar, and Brain Heart Infusion (BHI) Agar (all from Creative Microtech Corp., Taiwan) and incubated at 30 °C for up to 2–4 weeks. Well-isolated colonies were then selected for further identification using VITEK 2 software (version R02.03) via VITEK 2 automated system (bioMérieux) with GN, GP, or YST identification cards, as appropriate. Susceptibility tests for levofloxacin and trimethoprim/sulfamethoxazole were carried out using a VITEK 2 automatic system, and breakpoint interpretation was aligned with the Clinical Laboratory Standard Institute CLSI guidelines for *S. maltophilia*.

### 2.3. Statistical Analyses

All results were analyzed using a commercially available software package (SPSS, version 21.0; SPSS Inc., Chicago, IL, USA). Continuous variables are described as medians and interquartile ranges (IQRs), and categorical variables are expressed as numbers and percentages. A comparison between survivors and non-survivors was made among patients with IAIs included in this study. The Kolmogorov–Smirnov test was used to assess the normal distribution of continuous variables. Continuous variables were compared using Student’s *t*-test or the Mann–Whitney U-test, as appropriate. Categorical variables were analyzed using the chi-square test. A multivariable logistic regression analysis was used to identify factors independently associated with in-hospital mortality. Variables with a *p* value < 0.1 after univariate analysis were entered into the multivariable model to identify independent risk factors for in-hospital mortality in patients with *S. maltophilia* IAIs, with the adjusted odds ratio (OR) and 95% confidence intervals (CIs) reported. All *p*-values were two-tailed, and a *p*-value less than 0.05 was considered statistically significant.

## 3. Results

**Figure 1:**
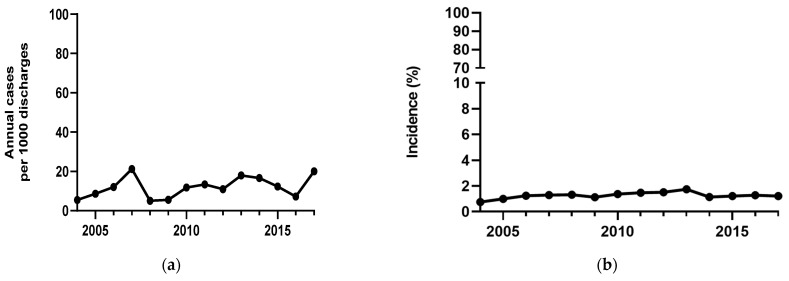
(**a**) Annual incidence trend (cases per 1000 discharges per year) of *S. maltophilia* infection/colonization from 2004 to 2017; (**b**) the annual incidence trend of IAIs among patients with *S. maltophilia* infection/colonization from 2004 to 2017. IAIs, intra-abdominal infections.

During the study period, the annual incidence of *S. maltophilia* infections/colonizations per 1000 patient discharges from 2004 to 2017 ranged from 5 to 20 cases, as shown in Figure 1a. During the same period, the incidence of IAIs due to *S. maltophilia* among patients with *S. maltophilia* colonizations/infections ranged from 0.5% to 2%, as shown in Figure 1b. The incidence of IAIs caused by *S. maltophilia* remained stable throughout the study period.

**Figure 2:**
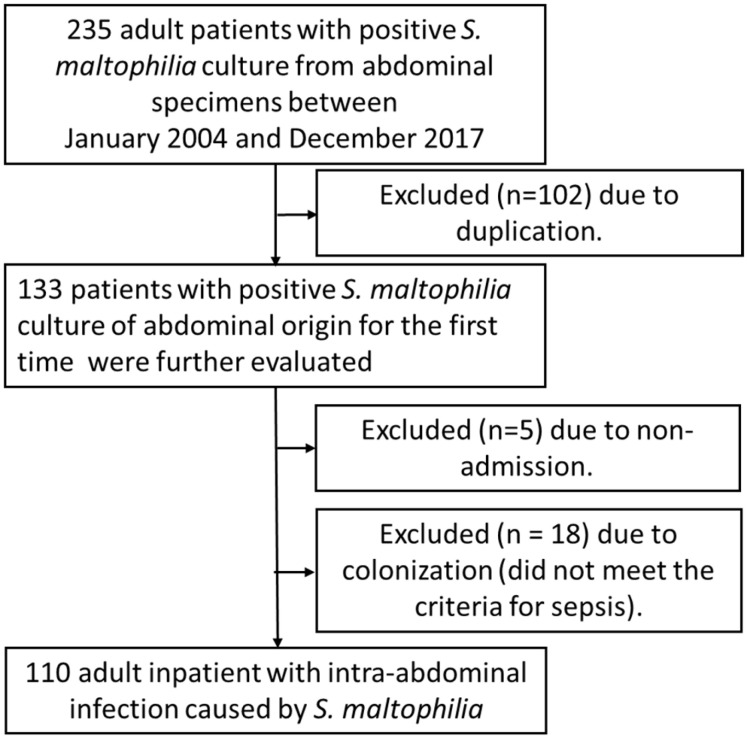
Flow diagram of patients with *S. maltophilia*–related intra-abdominal infections.

Based on the predefined inclusion and exclusion criteria, a total of 110 adult inpatients with IAIs caused by *S. maltophilia* were included in the final analysis after exclusion. A flow diagram for patient identification is shown in Figure 2. Among the 110 cases, 43 (39.1%) were from medical wards, 42 (38.2%) from surgical wards, and 25 (22.7%) from intensive care units; no cluster cases were identified.

Baseline characteristics of patients with IAIs due to *S. maltophilia* are shown in Table 1. Overall, the cohort consisted predominantly of older male patients with high comorbidity burdens, with most infections acquired in healthcare settings. The median Charlson Comorbidity Index was 6.5, reflecting significant underlying disease.

Malignancy was the most common comorbidity, particularly gastrointestinal (GI) cancers. Among patients with a malignancy (*n* = 62), the vast majority (87.1%) had GI cancers. Liver cancer was the most common subtype (*n* = 26), followed by bile duct (*n* = 9), stomach (*n* = 8), colorectal (*n* = 5), gallbladder (*n* = 4), and pancreatic cancer (*n* = 2). The remaining non-GI malignancies (12.9%) included lung cancer (*n* = 2), bladder or kidney cancer (*n* = 3), cervix cancer (*n* = 1), and lymphoma (*n* = 2).

Biliary tract infections and abdominal surgery-related infections were the leading causes of IAIs. At disease onset, the median SOFA score among patients was 4, and neutropenia was observed in only one patient (0.9%). Most patients had received prior antibiotics (94.5%), including substantial proportions of carbapenems (39.1%). Appropriate antibiotic treatment targeting *S. maltophilia* was administered within 48 h in only 15 (13.6%) patients. Infection control measures, including drainage procedures or surgical debridement, were frequently performed in addition to antibiotic therapy (74.5%). Among the 82 patients who received infection control measures, 22 received surgical debridement, and others (*n* = 60) received a drainage procedure. The 30-day mortality rate was 12.7% (*n* = 12), while the in-hospital mortality rate was significantly higher at 45 patients (40.9%). Among the 65 surviving patients, the median length of hospital stay after *S. maltophilia*-related IAI onset was 15 days. A comparison of patients who died and survived during hospitalization from *S. maltophilia*-related IAIs is also provided in Table 1. Several significant risk factors for in-hospital mortality were identified, including a longer hospital stay before isolation, an underlying malignancy, a higher Charlson comorbidity index, spontaneous bacterial peritonitis, a higher SOFA score, and shock (all *p* < 0.05). Immediate antibiotic targeting for *S. maltophilia* within 48 h of sepsis onset was a protective factor for mortality with borderline statistical significance (*p* = 0.065).

Among the 110 *S. maltophilia* isolates, 87 (79.1%) were susceptible to trimethoprim–sulfamethoxazole (TMP/SMX), while 23 (20.9%) were resistant. For levofloxacin, 88 isolates (80.0%) were susceptible, 2 (1.8%) were intermediate, and 20 (18.2%) were resistant. Notably, 11 isolates (10.0%) were resistant to both TMP/SMX and levofloxacin. Polymicrobial infections were observed in 84 (76.4%) patients. Only three (2.7%) patients had concurrent *S. maltophilia* bacteremia. In total, 140 concomitant pathogens isolated from abdominal specimens were observed, as shown in Table 2. Aerobic bacteria accounted for 80.7% (*n* = 113), with the top five most frequently isolated aerobic bacteria being *Enterococcus* spp. (*n* = 25), *Pseudomonas* spp. (*n* = 15), *Escherichia coli* (*n* = 12), *Acinetobacter* spp. (*n* = 10), and *Chryseobacterium indologenes* (*n* = 8). Isolated bacteria belonging to *Enterobacterales* (*Citobacter freundii*, *Enterobacter cloacae*, *Escherichia coli*, *Klebsiella pneumoniae*, and *Serratia marcescens*) comprised 19.3% (27/140) of the total isolates, while non-fermentative aerobic bacteria (*Achromobacter xylosoxidans*, *Acinetobacter* spp., *Burkholderia cepacia*, *Chryseobacterium indologenes*, *Chyseobacterium meningosepticus*, *Delftia acidororans*, *Pseudomonas* spp., *Rhizobium radiobacter*, *Sphingomonas paucimobilis*) represented 29.3% (41/140) of all isolates. Anaerobic bacteria and fungi were also identified, accounting for 9.2% (13/140) and 10% (14/140), respectively, with *Bacteroides* spp. (*n* = 10) and *Candida* spp. (*n* = 14) being the most common.

In the univariate analysis (Table 3), the presence of a malignancy (*p* = 0.011), spontaneous bacterial peritonitis (*p* = 0.019), shock (*p* < 0.01), and a SOFA score (*p* < 0.01) were significantly associated with mortality. Appropriate antibiotic targeting for *S. maltophilia* within 48 h led to increasingly improved outcomes (*p* = 0.089). In the multivariate logistic regression model, three independent predictors of mortality were identified: Appropriate antibiotic administration within 48 h was protective (OR: 0.168, 95% CI: 0.034–0.837, *p* = 0.029), while higher SOFA scores were associated with an increased risk of mortality (OR: 1.312, 95% CI: 1.109–1.552, *p* = 0.002). Additionally, malignancy remained a strong independent risk factor for mortality (OR: 4.533, 95% CI: 1.585–12.394, *p* = 0.005).

## 4. Discussion

*S. maltophilia* is considered an emerging nosocomial pathogen causing invasive infections among immunocompromised inpatients [1]. Bloodstream infections and respiratory tract infections caused by *S. maltophilia* have been widely studied, with various at-risk populations identified in the literature [19]. Additionally, an underlying hematological malignancy is commonly present in patients with *S. maltophilia* bacteremia [20,21]. The source of bacteremia is frequently associated with an infected central venous catheter, and prompt removal of the catheter, in combination with appropriate antibiotic therapy, has been shown to reduce mortality [22,23]. In contrast, *S. maltophilia*-related respiratory infections often occur in patients with chronic lung disease or in critically ill patients receiving care in intensive care units [24,25]. IAIs have not been well studied in previous research. Aside from several case reports, only two cohort studies partly describing IAIs caused by *S. maltophilia* have been previously published. One study by TL. Lin et al. focused on patients with IAIs following abdominal surgery, revealing that patients with IAIs caused by *S. maltophilia* had a higher mortality rate than those with infections from other pathogens. Other causes leading to IAIs, apart from abdominal surgery, were not mentioned in that study [26]. Another study by Ru Ma et al. evaluating the prognosis of patients with positive cultures of *S. maltophilia* from different sources also showed that *S. maltophilia* isolates of abdominal origin led to high mortality among infected patients. The lack of a distinction between infection and colonization in that study limited interpretation of the results [27]. Despite certain limitations, the results from both studies indicated a substantial impact of *S. maltophilia* on outcomes among patients with IAIs and should not be overlooked.

Our study is the first to comprehensively describe IAIs caused by *S. maltophilia* in the literature. In contrast to previous studies that focused upon *S. maltophilia* bacteremia or respiratory tract infections, our cohort examined patients with IAIs, revealing several significant findings. Similarly to patients with *S. maltophilia* bacteremia, malignancy was the most common underlying condition. However, gastrointestinal malignancies predominated, whereas hematologic malignancies and neutropenia were rare. Moreover, chronic lung diseases were less prevalent in our cohort, and only 25 patients (22.7%) were in intensive care units at the time of IAI onset. We also found that biliary tract infections, spontaneous bacterial peritonitis, and recent abdominal surgery accounted for the majority of cases. Several case reports in the literature have described different *S. maltophilia* abdominal infection sites, including liver abscesses [28], pancreatitis [29], continuous ambulatory peritoneal dialysis-related peritonitis [30], enterocolitis [31], and intra-abdominal abscesses following abdominal surgery [26]. Besides IAIs related to recent abdominal surgery, two other major causes of *S. maltophilia* IAIs in our study were biliary tract infection and spontaneous bacterial peritonitis, which have not been described previously. *S. maltophilia* can be isolated from a wide variety of aquatic or humid environmental sources, as well as medical equipment in hospital settings [19]. Therefore, IAIs related to recent abdominal surgery may result from contaminated surgical instruments or the hospital environment during the operation as reported [13]. However, for patients with biliary tract infections and spontaneous bacterial peritonitis caused by *S. maltophilia* in the current study, no recent abdominal surgery was noted. In addition to being isolated from environmental aqueous-associated sources, several reports have shown that *S. maltophilia* can be isolated from human feces, suggesting its ability to colonize the gastrointestinal tract, particularly in cancer patients [12,32]. Although we did not perform stool cultures on the patients in the present study, the high prevalence of malignancy among them—known to be a risk factor for *S. maltophilia* gastrointestinal colonization—may contribute to subsequent invasive IAIs, as previously reported [31]. Therefore, we speculate that endogenous intestinal colonization by *S. maltophilia* is the most likely source of IAIs among our patients without a recent history of abdominal surgery. Taken together, these findings suggest that patients with *S. maltophilia* IAIs represent a distinct clinical entity that differs from the classical paradigm of bacteremia in cancer patients or pneumoniae in critically ill patients. Our study has important implications for early recognition, microbiologic workup, and empirical therapy in this emerging subgroup.

Polymicrobial infections accounted for the majority of cases in the present study. A wide range of copathogens, in addition to *S. maltophilia*, were isolated from abdominal specimens. These included Gram-positive and Gram-negative bacteria, both aerobic and anaerobic, as well as yeast. Non-fermenting bacteria accounted for 29.3% of isolates, while *Enterobacterales* accounted for 19.3%. These findings differed from those in previous reports, which indicated that *Enterobacterales* accounted for nearly 80% of isolates obtained from patients with IAIs [11]. This discrepancy may be attributable to differences in study populations since our study population focused on the *S. maltophilia* IAI subgroups, rather than overall IAI cases. Generally speaking, patients with *S. maltophilia* infections had previously been exposed to broad-spectrum antibiotics, which may be selected for more non-fermenting pathogens and even fungi, as shown in our results [1,33]. Since IAIs caused by *S. maltophilia* presented a more diverse distribution of co-pathogens compared to overall IAIs, the early application of broad-spectrum antibiotics and even antifungal agents should be considered to cover multiple drug-resistant pathogens and fungi.

Only 2.7% (3/110) of patients with *S. maltophilia* IAIs had concurrent bacteremia, yet the overall mortality was as high as 40.9%. These results were consistent with those in a previous study demonstrating that *S. maltophilia* had a significant impact on mortality among patients. Further analysis revealed that patients with high SOFA scores and an underlying malignancy were associated with a high risk of mortality, indicating that disease severity and host factors played a significant role in mortality. This result is consistent with previous results from patients with *S. maltophilia* infections [10]. Notably, immediate antibiotic use targeting *S. maltophilia* within 48 h was associated with improved outcomes even though only 15 (13.6%) received immediate antibiotic use targeting *S. maltophilia*. Although *S. maltophilia* had low virulence and was usually co-cultured with other pathogens, our results suggest that patients still benefit from immediate targeted antibiotic use and should not be disregarded when isolated alongside other pathogens from abdominal specimens.

As the first study to describe the clinical characteristics of patients with IAIs caused by *S. maltophilia*, several limitations warrant consideration. First, the retrospective design of the study may have introduced selection and recall biases. Second, given that the study spanned approximately 14 years, changes over time in routine infectious disease care—such as changes in empirical antimicrobial strategies, the adoption of advanced microbiological diagnostics, and updates in clinical practice guidelines—may have impacted patient outcomes analysis. Third, differentiating between colonization and true infection is challenging due to the limited pathogenicity of *S. maltophilia*. Although only patients who met the sepsis criteria were included in this analysis, colonization could not be entirely ruled out, which may have introduced bias. Fourth, other concomitant pathogens were frequently isolated from patients with IAIs due to *S. maltophilia*, making it difficult to clearly define the pathogenic role of *S. maltophilia* and its impact on mortality. Finally, the results were derived from a single-center study, which limited the generalizability of the findings to institutions in other geographical regions.

IAIs caused by *S. maltophilia* were less frequent than those caused by *Enterobacterales* [11]. In our study, these infections were observed primarily among inpatients with gastrointestinal malignancies, liver cirrhosis, or recent abdominal surgery and were associated with high overall mortality, particularly among patients with underlying malignancies and elevated SOFA scores. Prompt initiation of targeted antibiotic therapy against *S. maltophilia* in at-risk patients with IAIs is crucial for improving clinical outcomes. Given the limitations of our study, a multicenter study with a larger sample size is urgently needed to validate our findings.

## Figures and Tables

**Table 1:** Clinical characteristics and a bivariable analysis of risk factors associated with in-hospital mortality among patients with intra-abdominal infections (IAIs) caused by *S. maltophilia*.

Variables	Total (*n* = 110)	Survivors (*n* = 65)	Non-Survivors (*n* = 45)	*p*-Value
Age, years ^a^	60 (49–69)	58 (48.5–68.5)	61 (51.5–71.0)	0.275
Male, *n* (%)	76 (69.1)	45 (69.2)	31 (68.9)	0.970
Community-acquired, *n* (%)	3 (2.7)	2 (3.1)	1 (2.2)	1.000
Hospital stay before isolation ^a^	16 (4–32.25)	13 (3.5–24)	26 (8–43)	0.005
Underlying disease, *n* (%)				
Cerebrovascular accident	15 (13.6)	9 (13.8)	6 (13.3)	0.939
Heart failure	8 (7.3)	3 (4.6)	5 (11.1)	0.179
Chronic obstructive pulmonary disease	4 (3.6)	4 (6.2)	0 (0)	0.117
Diabetes Mellitus	30 (27.2)	15 (23.1)	15 (33.3)	0.166
Chronic kidney disease ^b^	27 (24.5)	16 (24.6)	11 (24.4)	0.584
Peptic ulcer disease	38 (34.5)	19 (29.2)	19 (42.2)	0.114
Malignancy	62 (56.3)	30 (46.2)	32 (71.1)	0.008
Liver cirrhosis	39 (35.5)	20 (30.8)	19 (42.2)	0.151
Charlson comorbidity index ^a^	6.5 (4–8)	5 (3–8)	7 (6–8.5)	0.012
Causes of intra-abdominal infection, *n* (%)				
Biliary tract infection	47 (42.7)	30 (46.2)	17 (37.8)	0.383
Abdominal surgery-related	39 (35.4)	26 (40.0)	13 (28.9)	0.231
Spontaneous bacterial peritonitis	22 (20.0)	8 (12.3)	14 (31.1)	0.015
Others ^c^	2 (1.8)	1 (1.5)	1 (2.2)	1.000
Previous antibiotic use before IAI onset, *n* (%)	104 (94.5)	62 (95.4)	42 (93.3)	0.687
Polymicrobial infection, *n* (%)	84 (76.4)	47 (72.3)	37 (82.2)	0.165
Disease severity, *n* (%)				
SOFA score ^a^	4 (2–8)	3 (1–5)	7 (4–9.5)	<0.001
Neutropenia, *n* (%)	1 (0.9)	0 (0)	1 (2.2)	0.409
Septic shock, *n* (%)	23 (20.9)	5 (7.7)	18 (40.0)	<0.01
Appropriate antibiotic targeting for *S. maltophilia* within 48 h, *n* (%)	15 (13.6)	12 (18.5)	3 (6.7)	0.065
Infection control measures, *n* (%)	82 (74.5)	49 (75.4)	33 (73.3)	0.808

SOFA, Sequential Organ Failure Assessment. ^a^ Data are presented as median (interquartile range). ^b^ Blood creatinine levels above 2 milligrams per deciliter (mg/dL). ^c^ One case was related to continuous ambulatory peritoneal dialysis-associated. peritonitis; the other was due to a perforated colon.

**Table 2:** Distribution of concurrent pathogens in patients with an intra-abdominal infection due to *S. maltophilia*.

Pathogen	Total (*n* = 140)
Gram-positive aerobic bacteria	
*Enterococcus* spp.	25
*Lactobacillus* spp.	1
*Leuconostoc* spp.	1
*Coagulase-negative staphylococci*	7
*Staphylococcus saprophyticus*	2
*Staphylococcus aureus*	5
*Viridans streptococci*	2
**Subtotal**	**43**
Gram-positive anaerobic bacteria	
*Clostridium sporogenes*	1
*Peptostreptococcus magnus*	1
**Subtotal**	**2**
Gram-negative aerobic bacteria	
*Achromobacter xylosoxidans*	2
*Acinetobacter* spp.	10
*Aeromonas hydrophila*	2
*Burkholderia cepacia*	1
*Burkholderia multivorans*	1
*Chryseobacterium indologenes*	8
*Chryseobacterium meningosepticus*	1
*Citrobacter freundii*	1
*Delftia acidovorans*	1
*Enterobacter cloacae*	5
*Escherichia coli*	12
*Klebsiella pneumoniae*	5
*Pseudomonas* spp.	15
*Rhizobium radiobacter*	1
*Serratia marcescens*	4
*Sphingomonas paucimobilis*	1
**Subtotal**	**70**
Gram-negative anaerobic bacteria	
*Bacteroides* spp.	10
*Fusobacterium nucleatum*	1
**Subtotal**	**11**
Fungi	
*Candida* spp.	14
**Subtotal**	**14**

**Table 3:** Logistic regression analysis of risk factors for in-hospital deaths under intra-abdominal infection with *S. maltophilia*.

Variable	Unadjusted OR (95% CI)	*p*-Value	Adjusted OR (95% CI)	*p*-Value
Age	1.013 (0.989–1.038)	0.299		
Sex	0.984 (0.433–2.239)	0.970		
Underlying disease				
Cerebrovascular accident	0.957 (0.315–2.907)	0.939		
Heart failure	2.583 (0.585–11.411)	0.210		
Diabetes Mellitus	1.667 (0.715–3.887)	0.237		
Chronic kidney disease	0.991 (0.410–2.397)	0.984		
Peptic ulcer disease	1.769 (0.797–3.927)	0.161		
Malignancy	2.872 (1.280–6.445)	0.011	4.433 (1.585–12.394)	0.005
Liver cirrhosis	1.644 (0.745–3.630)	0.219		
Causes of intra-abdominal Infection				
Biliary tract infection	0.708 (0.326–1.538)	0.383		
Abdominal surgery-related	0.609 (0.270–1.375)	0.233		
Spontaneous bacterial peritonitis	3.218 (1.217–8.510)	0.019	2.144 (0.666–6.897)	0.201
Disease severity				
SOFA score	1.298 (1.150–1.466)	<0.01	1.312 (1.109–1.552)	0.002
Shock	8.000 (2.690–23.793)	<0.01	2.302 (0.538–9.859)	0.261
Appropriate antibiotic targeting for *S. maltophilia* within 48 h	0.315 (0.084–1.191)	0.089	0.168 (0.034–0.837)	0.029
Infection control measures	0.608 (0.256–1.443)	0.259		

Further multivariate. 4 SOFA, Sequential Organ Failure Assessment; OR, odds ratio; CI, confidence interval.

## Data Availability

The study data are available upon reasonable request to the authors.

## Abbreviations

The following abbreviations are used in this manuscript:

IAI	Intra-abdominal infection
SOFA	Sequential Organ Failure Assessment
IQR	Interquartile range
OR	Odds ratio
CI	Confidence interval
